# Preparation of Nano/Microcapsules of Ozonated Olive Oil in Hyaluronan Matrix and Analysis of Physicochemical and Microbiological (Biological) Properties of the Obtained Biocomposite

**DOI:** 10.3390/ijms232214005

**Published:** 2022-11-13

**Authors:** Gohar Khachatryan, Lusine Khachatryan, Magdalena Krystyjan, Anna Lenart-Boroń, Marcel Krzan, Klaudia Kulik, Anna Białecka, Maja Grabacka, Nikola Nowak, Karen Khachatryan

**Affiliations:** 1 Faculty of Food Technology, University of Agriculture in Krakow, Mickiewicz Ave. 21, 31-120 Krakow, Poland; 2 Department of Orthopaedics and Traumatology, University Hospital in Cracow, Macieja Jakubowskiego 2, 30-688 Krakow, Poland; 3 Department of Microbiology and Biomonitoring, Faculty of Agriculture and Economics, University of Agriculture in Krakow, 30-059 Krakow, Poland; 4 Jerzy Haber Institute of Catalysis and Surface Chemistry, Polish Academy of Sciences, Niezapominajek 8, 30-239 Krakow, Poland; 5 Jan Bober Center for Microbiology and Autovaccines, 31-016 Krakow, Poland

**Keywords:** hyaluronic acid, nanocapsules, ozone, olive oil, rheology, in vitro

## Abstract

Hydrogels, based on natural polymers, such as hyaluronic acid, are gaining an increasing popularity because of their biological activity. The antibacterial effect of ozone is widely known and used, but the instability the gas causes, severely limits its application. Ozone entrapment in olive oil by its reaction with an unsaturated bond, allows for the formation of stable, therapeutically active ozone derivatives. In this study, we obtained an innovative hydrogel, based on hyaluronic acid containing micro/nanocapsules of ozonated olive oil. By combination of the biocompatible polymer with a high regenerative capacity and biologically active ingredients, we obtained a hydrogel with regenerative properties and a very weak inhibitory effect against both bacterial commensal skin microbiota and pathogenic *Candida*-like yeasts. We assessed the stability and rheological properties of the gel, determined the morphology of the composite, using scanning electron microscopy (SEM) and particle size by the dynamic light scattering (DLS) method. We also performed Attenuated total reflectance Fourier transform infrared (FTIR-ATR) spectroscopy. The functional properties, including the antimicrobial potential were assessed by the microbiological analysis and in vitro testing on the HaCat human keratinocyte cell line. The studies proved that the obtained emulsions were rheologically stable, exhibited an antimicrobial effect and did not show cytotoxicity in the HaCat keratinocyte model.

## 1. Introduction

The skin is the second largest organ of the body of vertebrates (after the gut). It has a complex structure and multiple functions, most importantly the isolation of the internal environment of the organism from the exterior, in particular the mechanical defense against pathogenic microorganisms. Just as any other organ, the proper functioning of the skin depends on its condition [1,2]. A disrupted skin barrier weakens the defense mechanisms, compromises the integrity and leads to changes in pH, dehydration and inflammation—it poses a serious threat to the health of the individual [3]. Thus, the materials used in providing additional protection to the skin or supporting the wound healing process, present an important therapeutic potential. In recent years, many types of dressings have been developed to enhance the tissue regeneration [4,5,6,7,8,9], but current scientific evidence shows that hydrogels are the most advantageous because they constantly provide a moist environment that is beneficial for the healing process [10,11,12,13].

Natural polymer-based hydrogels are gaining an increasing popularity because of their biological activity that imitates the natural extracellular matrix and supports the cell proliferation, adhesion and structural repair. What is more, they are biocompatible and non-toxic. Hyaluronic acid, which is ubiquitous in vertebrates (including humans), especially in the cartilage tissue and synovial fluid, occupies here a special place. It is involved in various biological processes, such as cell differentiation or embryonic development. It also reduces inflammation and promotes various stages of wound healing [14], playing different roles, depending on the particle size [15,16]. The high molecular weight of hyaluronic acid is broken down into low molecular weight oligomers, which promote the leukocyte chemotaxis and the expression of the immunomodulating mediators. In addition, it shows the anti-angiogenic and anti-inflammatory activities [17,18,19]. Acting as a membrane-forming polymer, it allows the tissue irrigation and takes part in providing an osmotic balance, thus contributing to the skin hydration and decreasing the transepidermal water loss [12]. These observations have encouraged the extensive investigation of hyaluronic acid properties. Hyaluronic acid-based hydrogels have successfully been used in complicated wound healing processes [12,17,18,20,21], they also found a place in aesthetic medicine or cosmetology [22,23,24].

Biofilm formation by the pathogenic bacteria is the main threat that hinders wound healing, it can hinder healing as well as cause an inflammation or, in critical cases, even lead to amputation. The prevention of biofilm formation by pathogenic bacteria is an important aspect of the regeneration of ruptured or damaged tissue, and has a crucial therapeutic importance.

Over the past few years, novel, intelligent materials with antimicrobial properties are gaining an increasing popularity [12].

Inspired by the most recent advances in biopolymer research, we decided to combine the unique properties of hyaluronic acid with ozone enclosed in olive oil. The antibacterial effect of ozone is widely known and used, but the instability of the gas, severely limits its application [25,26,27,28]. Ozone entrapment in olive oil, by its reaction with an unsaturated bond, allows for the formation of stable, therapeutically active ozone derivatives [29].

Not only ozone, but also olive oil, presents antimicrobial properties, especially against Gram—bacteria and fungi, such as Candida spp., which makes it an important ingredient for the topical application in skin inflammations or fungitis [30,31].

Olive oil ozonation allows to use the antibacterial potential of both of these ingredients. The bactericidal mechanism of ozone focuses on its oxidizing properties, which induce the destruction of cell walls and the bacterial plasma membranes. As a consequence, the membrane permeability increases and ozone penetrates into the bacterial cell [32,33].

In recent years, special attention has been paid to the use of nanomaterials in cosmetology and tissue engineering [34]. Their unique physicochemical properties allow to obtain long-lasting effects and increase the nanomaterial stability. The size of the nanomaterial allows a more efficient transport of the ingredients through the skin, directional action and pore penetration [35,36,37].

The aim of this study was to obtain an innovative hydrogel, based on hyaluronic acid, containing micro- and nanocapsules of ozonated olive oil and to assess its selected properties that are crucial for its potential applications: morphology, rheological properties and mechanical modulus of the gel, the microbiologial potential and cytotoxicity.

## 2. Results

**Figure 1:**
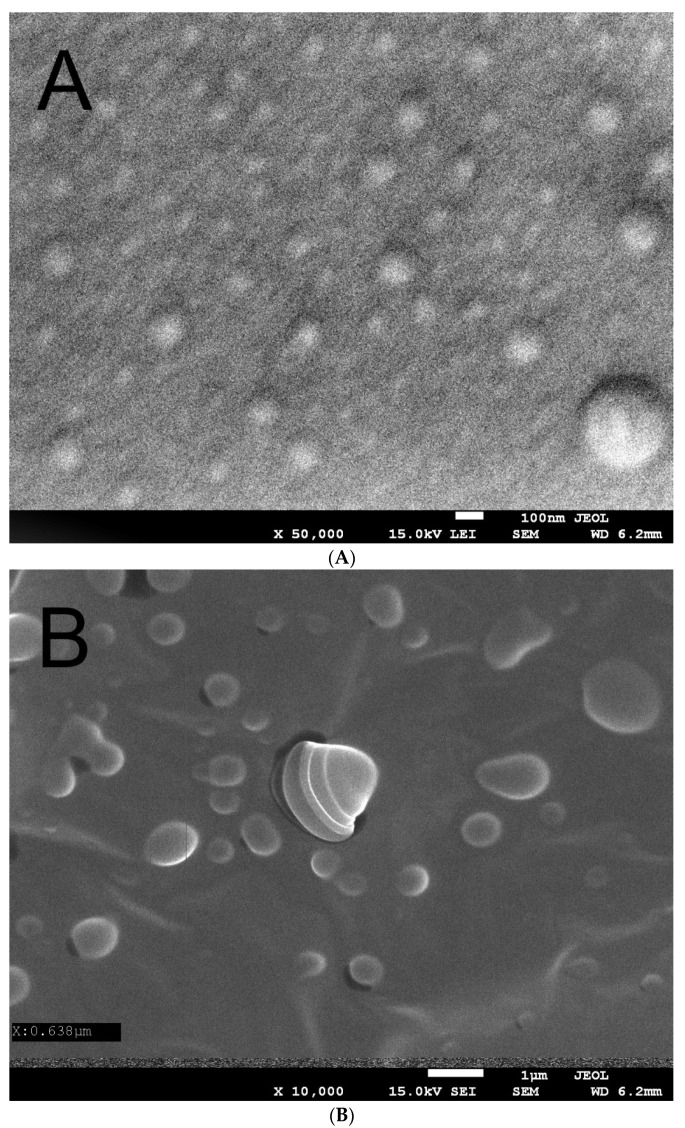
Scanning electron microscope image of the Hyal/O_3_ at ×50,000 (**A**) and ×10,000 (**B**) magnification.

Figure 1A,B shows the scanning electron microscope images of the Hyal/O_3_ film surface at 50,000 an 10,000 magnification, respectively.

**Figure 2:**
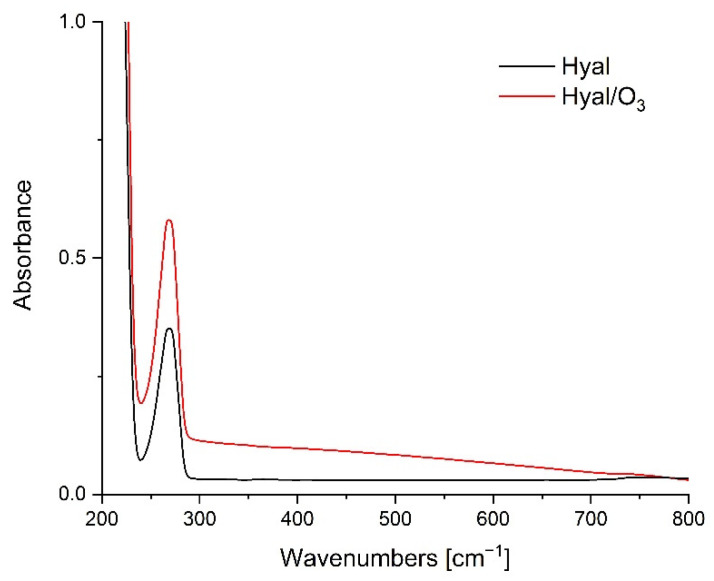
UV-Vis spectra for Hyal and Hyal/O_3_ films.

The UV-Vis spectra in the spectral range of 200–800 nm for the Hyal and Hyal/O_3_ films are presented on Figure 2.

**Figure 3:**
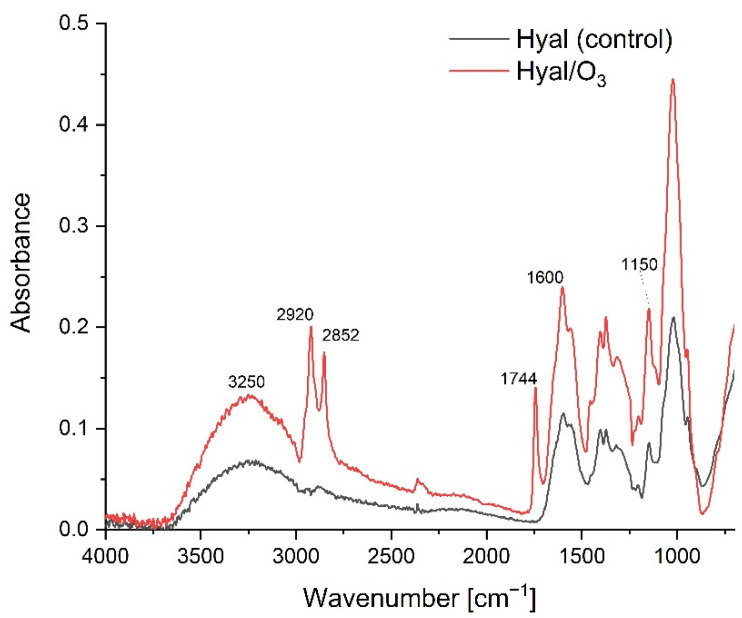
The FTIR-ATR spectra for the Hyal and Hyal/O_3_ films.

The FTIR-ATR spectra in the spectral range of 750–4000cm^−1^ for the Hyal and Hyal/O_3_ films are presented on Figure 3. The band for the oscillations of the 3600–2980 cm^−1^ can be attributed to the hydrogen-bonded O-H and N-H stretching vibrations of the N-acetyl side chain. A group of overlapping bands of moderate intensity is observed at approximately 2910 cm^−1^, due to the C-H stretching vibrations. The bands at 1620 and 1410 cm^−1^ can be attributed to the asymmetric (C=O) and symmetric (C-O) stretching modes of the planar carboxyl groups in the hyaluronate [38].

**Figure 4:**
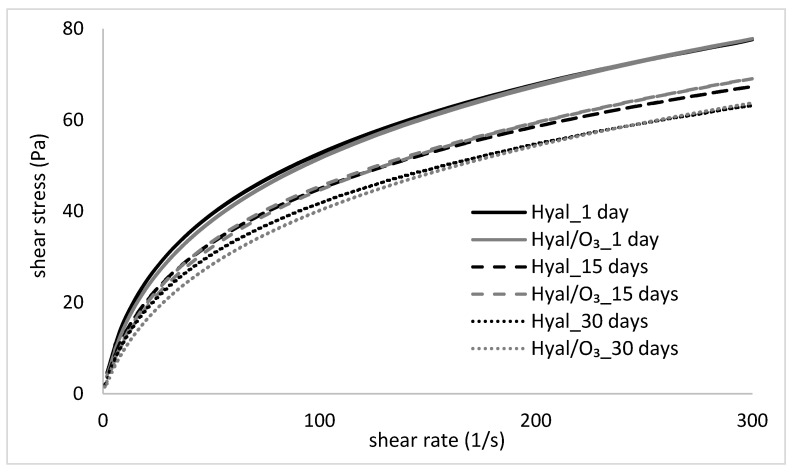
Flow cures of the Hyal and Hyal/O_3_ samples, measured at 1, 15 and 30 day of storage.

Figure 4 presents the changes in the flow curves of the hydrogels, stored for 30 days, while Table 1 shows the Ostwald de-Waele rheological model parameters, matched to the obtained curves. The values of the apparent viscosity at a specific shear rate are included in Table 2.

The flow curves of the examined hydrogels and nanoemulsions show a deviation from the Newtonian fluids and is typical for the pseudoplastic, shear-thinned fluids, which correspond with the literature data [39,40,41,42,43]. The shear stress rates of both examined samples (Hyal and Hyal/O_3_) are very close to each other and show a similar pattern of flow curves (Figure 4). In both cases, the changes related to the storage time of the samples were noticed—a gradual decrease of shear stress occurred, which is evidence of a decrease of the apparent viscosity. This is also confirmed by the parameters of the flow curves, because the consistency coefficient decreased with the storage time of the samples (Table 1). The differences in viscosity between the Hyal and Hyal/O_3_ samples on the same measurement day are small or statistically insignificant, especially at higher shear rates (Table 2). However, the changes caused by the storage time, turned out to be statistically significant. Comparing the consistency coefficient values for the Hyal gel, a decrease of this parameter was observed on days 15 and 30, compared to day 1. A similar trend was observed for the Hyal/O_3_ emulsion.

### 2.1. Frequency Sweep

**Figure 5:**
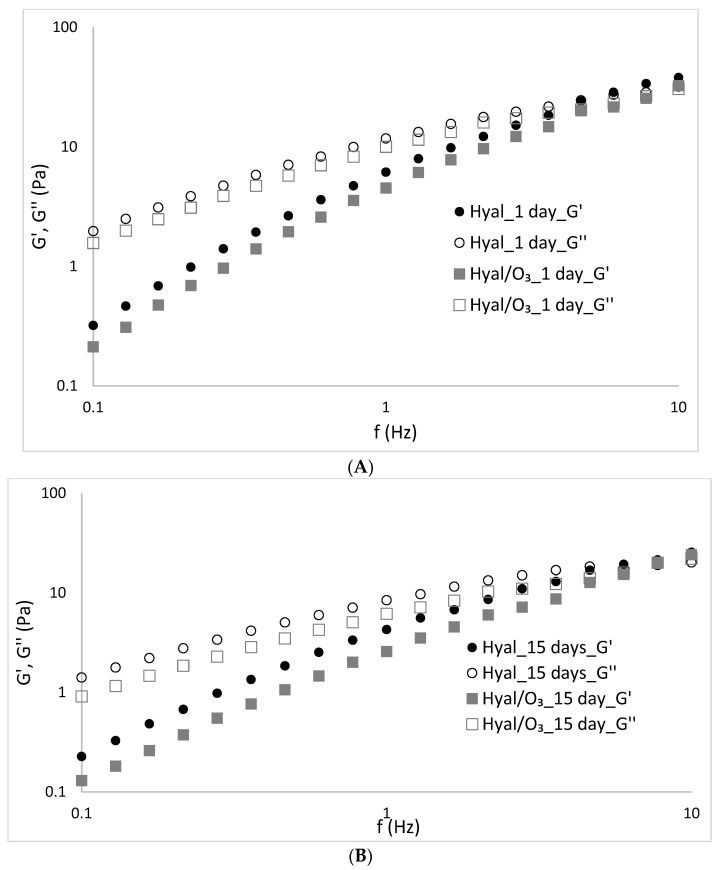

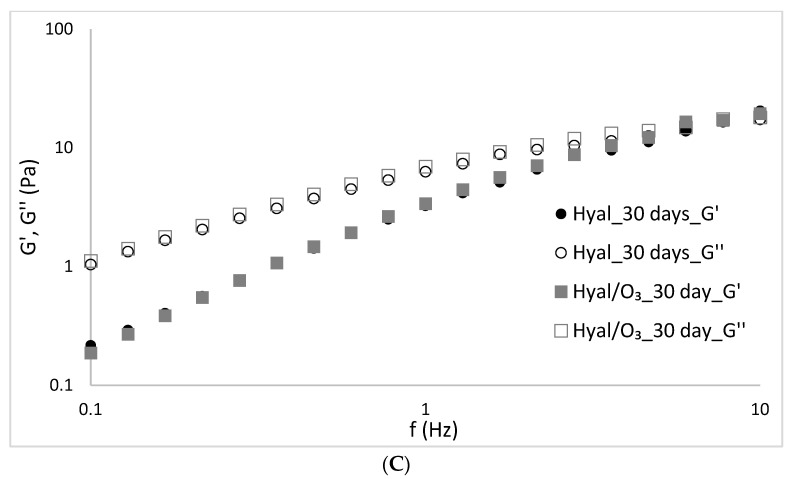
Values of the storage and loss moduli (G′ and G″) in the stress sweep test of the Hyal/O_3_ sample measured at (**A**) 1, (**B**) 15 and (**C**) 30 days of storage

The dependences of the elasticity and loss moduli vs the frequency are shown in the Figure 5.

### 2.2. Analysis of the Particle Size Distribution and Particle Charge

The obtained results show enormous differences between the samples. Hyal reference samples contain particles of the order of 4000 nm, while in the Hyal samples supplemented with ozone, the particles are four times smaller (1060 nm). We see similar significant differences in the zeta potential results, which for Hyal is −55 mV, and for the Hyal/O_3_ samples −81 mV (with the measurement error less than 1 mV).

### 2.3. Microbiological Analysis—The Effect of Hyal/O_3_ on the Microbial Growth

**Figure 6:**
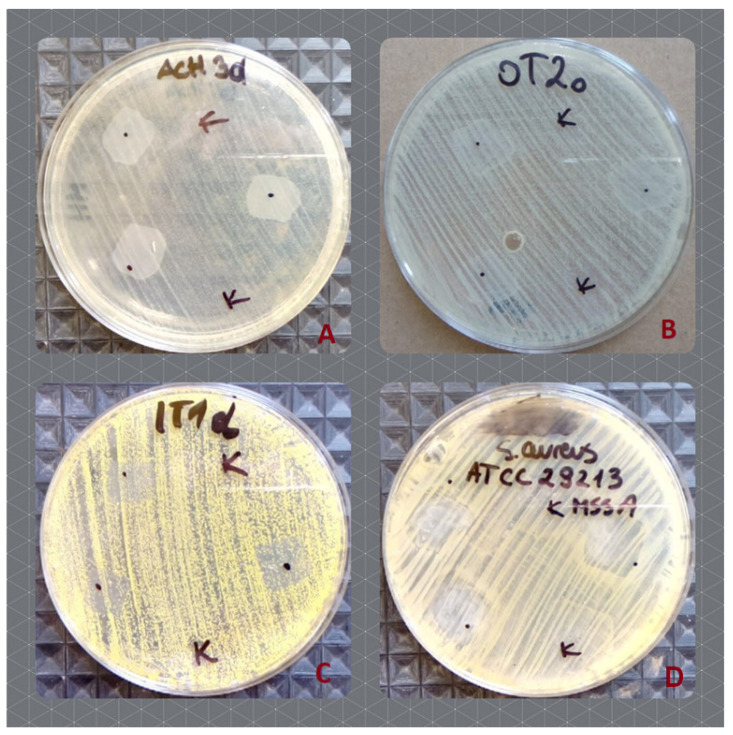
The effect of the Hyal/O_3_ (dots) and control Hyal (K) foils on the growth of the selected bacterial strains. (**A**)—increased growth of S. *epidermidis,* isolated from the hands; (**B**)—increased growth of *S. epidermidis,* isolated from the under eye region; (**C**)—inhibited growth of *M*. *luteus,* isolated from hands, (**D**)—inhibited growth of the type strain of methicillin susceptible *S. aureus* ATCC 29213.

Out of the examined 53 skin commensal bacterial strains, 17 showed a slight growth increase (Table 3, Figure 6A,B) as a result of the application of the Hyal/O_3_ foils. The growth of five bacterial strains was inhibited (Table 4, Figure 6C,D). In the case of the remaining 31 isolates, no effect was observed. Moreover, out of the 30 *Candida* strains, the growth of only five was inhibited (Table 4), while no effect was observed for the remaining 25 strains. The control foils (sole Hyal) caused no effect—neither an increase, nor an inhibition of the bacterial and *Candida* growth was observed in vitro (Table 4). The statistical analysis showed that the differences in the growth inhibition zones were statistically significant, only between *S. aureus* and *Candida* (H = 15.88; *p* = 0.0012), while for the varying origin of strains, the results differed significantly (H = 20.78; *p* = 0.0009) between the type strains and those isolated from the skin lesions (z = 3.4; *p* = 0.01) and between the type strains and the one isolated from the eye (z = 3.67; *p* = 0.004).

### 2.4. The Assessment of Cytotoxicity in the HaCat Keratinocyte Model

**Figure 7:**
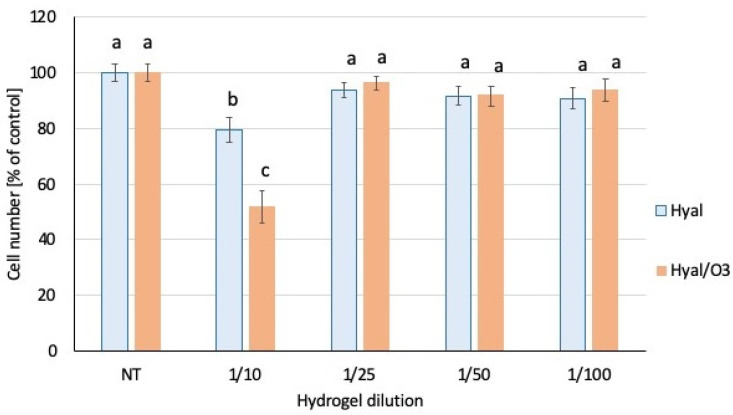
Number of viable cells in the culture. The bars represent the mean number of cells (*n* = 8) ± SD. Values denoted with the same letters do not differ statistically at the level of confidence *p* < 0.05.

Both hyaluronic acid-based hydrogels (with and without the ozonated olive oil nanocapsules) were very well tolerated by the HaCat keratinocytes, and the dilutions 1:25 and greater did not significantly affect the number of viable cells in the culture (Figure 7). Only in case of the highest tested concentration of both hydrogels (1:10 dilution), the incubation lead to the statistically significant reduction of the cell viability: 80% and 52% of viable cells for the hyaluronic hydrogel (Hyal) and the hyaluronic hydrogel enriched in the ozonated olive oil (Hyal/O_3_), respectively (Figure 7). No signs of cytotoxicity, such as abnormal morphology, floating detached cells or cell debris in the cell culture were observed, so the decreased cell number is likely a result of a slowed down proliferation rate, rather than cell death. The hydrogel that contained ozonated olive oil nanocapsules (Hyal/O_3_) exerted the stronger growth inhibiting effect, than the pure hyaluronic acid hydrogel (Hyal). The cell culture medium with the highest concentration of hydrogels (1:10) had an opaque, cloudy appearance and was slightly more viscous than a standard medium, so the observed inhibition of the proliferation could possibly be attributed to impeded gas or nutrient diffusion.

## 3. Discussion

The SEM microscopy showed that the spherical nanocapsules, sized 50–100 nm, with an active substance, can be observed, as well as the single capsules with dimensions of 150–200 nm (Figure 1a). This image confirms the presence of nanocapsules in the produced matrix. Figure 1b was performed at a longer exposition time, which caused the swelling and cracking of the capsules. We can observe bigger (swollen) structures, sized 100–1000 nm. Some capsules cracked during the analysis under the influence of the electron beam, showing their core-shell structure (core-ozonated olive oil, shell-hyaluronic acid). Similar structures have been observed in the case of composites containing ozonated olive oil in chitosan [44].

The Hyal spectrum shows a well-defined band at 269 nm, corresponding to the carbonyl groups of the hyaluronic acid molecule. The lack of a band shift may indicate the lack of interactions between the carboxyl group of Hyal and the ozonated oil. When such an interaction is present, we usually observe a bathochrome shift [45]. The spectra differ only in the higher absorbance of the Hyal/O_3_ sample, relative to the Hyal, which results from the lower transparency of the Hyal/O_3_ sample. The lack of differences may also indicate that the entire amount of the ozonated oil has been enclosed in capsules, which causes a reduction in the transparency and an increase in the absorption in the entire studied spectrum.

The addition of ozonated olive oil nanocapsules did not significantly affect the structural changes of the polymer (Figure 3). At the Hyal / O_3_ spectrum, we can see bands corresponding to the oil structure and an increase in the intensity of the absorption bands characteristic for oils. We can also observe vibrations corresponding to the methyl group in the range from 1350 to 1150 cm^−1^ that are the valence vibrations, corresponding to C-H in the -CH_3_ groups. The stretching vibrations of the C–O bonds belonging to esters consist of two coupled asymmetric vibrations C–C(=O)–O and O–C–C occur in the region between 1300–1000 cm^−1^. The bands coming from the C–C(=O)–O vibrations of the saturated esters are visible in the range of 1240–1163 cm^−1^, and in unsaturated esters, the vibrations are visible at lower wavenumbers. The vibration band of the O–C–C bond, which comes from the esters of the primary alcohols, appears in the region of 1064–1031 cm^−1^, and from the esters derived from the secondary alcohols at 1100 cm^−1^. Both types of esters are found in triacylglycerol molecules [46]. The low-intensity band at 1390 cm^−1^ comes from the C–H combination vibrations. The bands at 1726 cm^−1^ and 1760 cm^−1^ come from the first overtone of the C–H stretching vibrations of the methyl, methylene and ethylene groups. For oleic acid, a band is observed at 1725 cm^−1^, while saturated and trans-unsaturated triacylglycerols show absorption bands with a maximum at 1725 cm^−1^ and 1760 cm^−1^. The characteristic band at 2145 cm^−1^ comes from the C–C and the C–H stretching vibrations. Then several vibrations with a maximum at approx. 2952, 2921 and 2855 cm^−1^ come from valence -C-H vibrations from groups -CH_3_, CH_2_, respectively, in triglycerides [47].

The decrease in the viscosity of the hydrogels and nanoemulsions over time, and thus their thinning, may be a result of the weakening of the intermolecular interactions and indicate a slow destabilization of the formed structure. The obtained parameters are extremely important in the assessment of the properties of the gels and the emulsions intended for application on the skin, because they are related to a specific shear rate. According to the literature, cream spooning and pouring occurs at a shear rate of 10–100 s^−1^, while rubbing the cream on the skin occurs at a shear rate of as much as 1000 s^−1^ [41,48,49]. For comparison, Table 2 shows the viscosity values of the tested gel and the Hyal-based nanoemulsions for the individual shear rates. As can be seen from the presented data, the apparent viscosity of the preparations decreased with the increase of the shear rate, which proves their thinning. Moreover, when comparing the obtained results with the literature data [48,50], we observed that the apparent viscosity values of the tested systems, compared to the typical creams applied to the skin, were much lower and more similar to the rare, delicate, semi-solid systems, than the dense, heavy creams. The advantage of such systems is undoubtedly the ease of rubbing on the skin even at the low applied shear rates. This is also confirmed by the literature data, according to which the rheological properties of the product, especially the parameters, such as the yield stress and apparent viscosity in the lower shear rate ranges, can be correlated with the empirically subjective assessment of skin sensations (application and distribution of the preparation on the skin). Moreover, the apparent viscosity determined for the upper range of the shear rate (γ = 500 s^−1^) enables the final evaluation of spreading the sample on the skin, which increases with the decreasing viscosity [49,51,52].

By analyzing the obtained data (Figure 4), it was found that the tested gels and nanoemulsions showed a low flow limit. It is an important parameter in the assessment of the quality of the gels and emulsions that can be used in the production of medical ointments. A too high yield stress value indicates a heavy consistency of the product and difficulties in its distribution on the skin. From the consumer’s point of view, this is an undesirable feature as it may lead to skin irritation, which in turn discourages from the regular use. Moreover, emulsions with high values of yield stress are characterized by a lower efficiency [49,51,52].

An important parameter characterizing the nanoemulsions intended as creams and various types of healing ointments, is thixotropy, visible as hysteresis loops. The desired physical features, which the consumer pays close attention to, are the ease of application of the preparation to the skin. It is therefore important that the emulsion can return to its original shape when pressure is applied. The semi-solid product is applied to the skin by the force of the touch and transforms into a liquid. The emulsion must quickly re-bond and restore the semi-solid form, i.e., be thixotropic [50]. In the tests conducted in this study, the occurrence of a small hysteresis field was observed (Table 1). In the case of low-viscosity systems, i.e., more semi-solid than solid systems, it can be considered an advantage, because in this case, a too high thixotropy would indicate a significant dilution of the product during the shear and a slow return to its original state after the effect of shear forces has subsided, and such a behavior would make it difficult for the application and absorption of emulsions into the skin. It should also be noted that the reconstruction of the shear-damaged structure, in case the emulsion took place very quickly, may indicate its high stability, and no disintegration into a separate phase.

The frequency sweep test is performed in the LVR range, and is aimed not to destroy the structure, so that the measurements can provide information about the intermolecular forces present in the material [53]. The increase of the moduli values along the increase of frequency is observed for the whole frequency range. The Hyal and Hyal/O_3_ gels showed an advantage of viscous properties over elastic ones in the low frequency range 0.1–4.0, and at higher frequency values, a module intersection, above 4 Hz G′> G″ was observed. Such a course of curves proves the properties typical for concentrated solutions, showing a tendency for a more solid-like (gel-like) behavior at higher frequencies [54,55]. Only a slight decrease in the mechanical modulus (G′ and G″) was observed with the storage time of the Hyal gel and the Hyal/O_3_ emulsion.

To our best guess, hyaluronic acid in solutions without additives creates large flat structures. Meanwhile, when it interacts with the olive droplets, its structures are adapted to the shape and size of the olive droplet, which they tightly cover. Therefore, the particles of Hyal/O_3_ can be many times smaller than the particles of the hyaluronic acid itself. Increasing the negative surface potential of such shells is also logical, since −81mV is the real results of the negative surface potentials of hyaluronic acid and olive oil. The mechanism of interaction between these components remains unknown. More negative surface probably was formed due to the ozone oxidation. A similar behaviour was observed in the PLA materials after the photo-oxidation, due to the UV irradiation [56]. It was already proved that under the influence of ozone, the surface becomes functional, as a result of which the oxygen-containing functional groups are included of material interface [57]. The functional group oxidation is complicated and probably requires complicated pathways [58]. Authors also confirmed that the ozonation of solid materials can increase the specific surface area, because ozone reacts with the physical structure of the materials, enlarging the pore size and creating new pores. An increase in the pore structure was seen at the micropore level.

A few preliminary and pilot studies have been published on the effect of ozonated oils on pathogenic bacteria [59,60,61] and *Candida* yeasts [62,63,64,65], or both [66,67], as well as the possibility of their topical applications [33]. None of the studies published so far have dealt with the possible impact of ozonated oils on commensal skin microbiota. The results presented by various authors are either contrary, or show varying, sometimes disputable effects against microorganisms. For example, Pietrocola et al. [59] examined the effect of ozonized olive oil against Gram-positive and Gram-negative oral and periodontal pathogens. They observed a moderate antiseptic effect of this preparation, definitely lower, as compared to the classic chlorhexidine preparation. Silva et al. [61], based on the study involving the methicillin-resistant *S. aureus*, observed the growth inhibition caused by ozonated oils and suggested that their use is promising in the treatment of skin infections. Radzimierska-Kaźmierczak et al. [60] demonstrated a weak inhibitory effect of ozonated olive oil against *E. coli*, *S. aureus*, *C. albicans* and *Aspergillus brasilensis*, suggesting it to be a promising raw material for the cosmetics and pharmaceutical industries. The inhibitory effect against the skin microbiota and pathogenic *Candida*, observed in this study can also be assessed as weak to moderate. What is interesting, is that out of the 53 bacterial strains tested, the growth of 17 was slightly increased in vitro (Figure 6A,B), with the highest share represented by *Micrococcus luteus* (*n* = 10). No similar observations have been reported so far, therefore the ability of some bacterial strains to overcome the O_3_ treatment and to increase their growth should be subjected to further, more thorough, examinations. What is interesting, is that the growth of two other strains of *M. luteus* (isolated from hands) was inhibited, while the remaining five strains did not react to the application of the Hyal/O_3_ foils. Serio et al. [68] published a study examining the in vitro antibacterial effects of ozonated sunflower seed oil and observed a satisfactory growth inhibition of both Gram-negative and Gram-positive strains of bacteria, including *M. luteus*. As shown in Table 3, the Hyal/O_3_ foils caused a growth inhibition of the commensal bacteria and *Candida* yeasts, but the mean values of growth inhibition zones were higher for the bacteria than for *Candida* and the differences in the growth inhibition zones were statistically significant between *Candida* and *S. aureus*. In our study, the highest mean values of growth inhibition zones were observed for both type strains of *S. aureus* (methicillin-resistant -MRSA and methicillin-susceptible -MSSA) strains, the same as in the study by Silva et al. [61], who observed a high activity of ozonated oils against both MSSA and MRSA strains. Similar observations to our experiments were made by Nocuń et al. [66], in a study on the activity of ozonated olive oil against nine species of pathogenic and potentially pathogenic bacteria and fungi, including *S. aureus*, *E. coli* and *C. albicans*. They observed that the effective concentration of ozonated olive oil was much higher against *Candida* (1.6% vol) than for *S. aureus* (0.2% vol), and that the mean growth inhibition zone diameter was nearly two times smaller in *Candida* (14 mm) than in *S. aureus* (26.7). The decreased susceptibility of the *Candida* strains towards antifungal agents can be associated with the structure and composition of their cell wall, which is two-layered and is composed of a β-glucan-chitin skeleton, which is responsible for the strength and shape of the cell wall [69]. It contains mannans which have a low permeability and porosity thus affect the resistance of the cell wall to the antifungal agents [69]. In terms of different *Candida* species, no statistically significant difference was observed (H = 3.79; *p* = 0.28). Similarly, Monzillo et al. [65] studied the effect of ozonized gel against four *Candida* species and observed the antimycotic activity of this preparation, but without clear differences between the different species. Moreover, Berenji et al. [63] observed a decreasing susceptibility of the *Candida* species to ozonized olive oil, in the following order *C. krusei* > *C. glabrata* > *C. albicans*. Furthermore, Nocuń et al. [66] also observed a significantly lower susceptibility of Gram-negative bacteria (*E. coli*) to ozonized olive oil than the Gram-positive *S. aureus*. This observation is also similar to the one in our study, but here we observed no growth inhibition of neither of the two type strains of *E. coli*. Nocuń et al. [66] attributed the lower susceptibility of Gram-negative bacteria to the ozonized preparations and antibiotics, to the presence of the outer lipopolysaccharide membrane and its decreased permeability [70]. Previous studies on hyaluronate hydrogels revealed a significantly impeded diffusion of various solutes in such gels, and the effect was proportional to the increase of the hyaluronate concentration and the molecular size of the solutes, as well as the presence of crosslinking agents [McCabe and Laurent 1975; Ogston and Sherman 1961; Kodavaty and Deshpande 2021]. For example, the diffusion of serum albumin and glucose in the hyaluronate solution (0.8 mg/mL) were reduced by 20- and approximately three fold, respectively [71], whereas more a recent study reported a 1.5–3.5 fold slower diffusion of fluorescein as a tracer molecule in 5% hyaluronate hydrogels crosslinked with divinyl sulfone, depending on the pH of the solution [72]. The diffusion of oxygen in hyaluronate gels (solidified with 6% agarose) was decreased by 7% [73]. These data suggest that the diffusion of nutrients (e.g., lipids), as well as the waste metabolites could be impaired in our cell cultures with the highest concentration of hydrogels (1:10). Particularly, the large proteins present in the serum-containing media, such as the growth factors necessary for sustaining the cell proliferation, could have a sub-optimal access to the keratinocytes, which resulted in a slower growth rate, as compared to the control cultures. However, our two-dimensional cell culture model has some limitations: in this model the cells have access to the nutrients, the growth factors and oxygen, only from the apical surface, whereas in the physiological condition in skin, the viable epidermis (stratum basale), the cells receive the necessary nutrients through the capillary vessel circulation. Therefore, the topical application of hyaluronate hydrogels (with or without ozonated olive oil) would not affect the nutrition or respiration of the keratinocytes in their microenvironment.

Hyaluronan facilitates the wound healing processes and supports the keratinocyte functions [74]. Our results are similar to those presented in other studies on non-crosslinked hyaluronic acid hydrogels, that did not show cytotoxicity in the Hacat model (cell viability remained 70% or greater) [74,75]. However, the concentrations tested in the latter study were much lower than in our experiments (0.01–0.5% vs. 1:10–1:100 in our study).

Hyaluronan promotes the keratinocyte proliferation and differentiation into corneocytes, which are terminally differentiated keratinocytes present in the stratum corneum, the external protective layer of the skin [76,77]. The formation of corneocytes is an important process, crucial for the development to the epidermal barrier. The molecular mechanism of the hyaluronan action involves binding to the surface glycoprotein receptor CD44 on the keratinocytes and the subsequent activation of the transcriptional program necessary for both the proliferation and differentiation [78]. The activation of CD44 by hyaluronan, induces the expression of cyclin D, involucrin, profilaggrin and cytokeratin 10 [76,77].

Hyaluronan and ozonated lipid mixtures have not been tested in HaCat cultures, but some studies demonstrated that Ozodrop^®^ preparations, containing ozonated sunflower oil liposomes with the addition of Hypromellose, stimulated the HaCat proliferation and expression of the antimicrobial peptides, such as calprotectins and calregulin C [79]. Ozodrop^®^ efficiently inhibited the expression of the proinflammatory cytokine CCL20 in keratinocytes, which might support the regeneration and alleviation of the local inflammation. Regenerative processes were further stimulated by the elevated expression of the migration markers, such as matrix metalloproteinases MMP2 and MMP9 by Ozodrop^®^, and the acceleration of the wound healing in a scratch wound assay by Ozodrop^®^ gel [79]. These results confirm the positive impact of the ozonated lipids on the skin physiology and suggest further direction of experimental analyses on the Hyal/O_3_ hydrogel to verify its activity, as well.

## 4. Materials and Methods

High molecular hyaluronic acid (Aquajuv CT), molecular weight 0.8–1.0 MDA; ozonated olive oil (Scandia Cosmetics S.A., Niepołomice, Polska) with an ozone content of 1.11 ± 0.02 g in 100 g of oil were used to produce the nanocomposite; microbiological media: Sabouraud Dextrose Agar (Graso Biotech, Jabłowo, Poland), CandiSelect agar (Bio-Rad, Marnes-la-Coquette, France), UTI Brilliance agar (Oxoid, Ceshire, UK), Columbia Agar with Sheep Blood Plus (Oxoid, Ceshire, UK), Baird-Parker agar (Biomaxima, Lublin, Poland), Mannitol Salt agar (Biomaxima, Lublin, Poland), Trypticase Soya Agar (Biomaxima, Lublin, Poland), Mueller–Hinton agar (Biomaxima, Lublin, Poland).

### 4.1. Determination of the Ozone Content

The ozone content in the vegetable oils used and the obtained products was determined by the peroxidation number, according to the procedure described in the European Pharmacopoeia [80].

### 4.2. Preparation of Ozonated Olive Oil Nanoemulsion

Nanoemulsion was prepared by placing a mixture of 5.0 mL of water and 5.0 mL of ozonated olive oil in an ultrasonic cleaner (Polsonic, Warsaw, Poland) and was sonicated for 30 min to obtain a nanoemulsion.

### 4.3. Hyaluronic Acid Hydrogel Preparation

1000.0 g of a 2% solution was prepared by weighing out 20.0 g of hyaluronic acid on an analytical weight (Radwag, Białystok, Poland) and afterwards supplementing it with 980.0 mL of deionized water. The resulting suspension was stirred using a magnetic stirrer (Heidolph RZR 2020, Heidolph Instruments GmbH & Co. KG, Schwabach, Deutschland) until a clear gel was obtained.

### 4.4. Samples Preparation

The previously obtained emulsion (10 mL) was slowly dropped into the 500 g hyaluronic acid gel, cooled down to 5 °C, while homogenizing. (Polytron PT 2500 E, Kinematica AG, Malters, Switzerland). A stable emulsion was obtained. For the SEM, FTIR-ATR oraz UV-VIS analysis, 100 g portions of gel were poured onto sterile 12 cm diameter polypropylene dishes and dried at room temperature to obtain the foils.

### 4.5. SEM Microscopy

The size and morphology of the nanoparticles thus prepared were analysed using a JEOL 7550 (Akishima, Tokyo, Japan) scanning electron microscope. Prior to the measurement, the prepared samples were sprayed (K575X Turbo Sputter Coater, Quorum Technologies Ltd, Lewes, UK) with 20 nm of chromium to increase a conductivity of the samples.

### 4.6. UV-VIS

UV-Vis absorption spectra of the composite obtained were analysed in the range of 200–800 nm using a Shimadzu 2101 (Shimadzu, Kyoto, Japan) scanning spectrophotometer.

### 4.7. FTIR-ATR

The FTIR-ATR spectra of the composite was analyzed using a MATTSON 3000 spectrophotometer (Madison, WI, USA). in the range of 4000–700 cm^−1^ with a resolution of 4 cm^−1^.

### 4.8. Rheological Measurements

Rheological measurements were determined using a RheoStress RS 6000 (Thermo Scientific, Karlsruhe, Germany) rotary rheometer equipped with a plate—plate P 35 Ti geometry. The temperature of the baseplate was 25.0 ± 0.1 °C. The measurement was carried out at freshly prepared samples (1 day) and after 15 and 30 days of storage in the fridge at 8 °C. On the measurement day, the sample was removed from the refrigerator and incubated at 25 °C for 1 h. The measurements were run in duplicate.

*Flow curves:* The shear rate was raised from 0.1 to 300 s^−1^, over a 10 min period and a subsequent decrease of the shear rate from 300 to 0.1 s^−1^, over a 10 min. The obtained flow curves were described by the Herschel–Bulkley rheological model:

$$\tau =\tau_{0\;}+K\cdot \;{\dot{\gamma }}^{n}$$

where: *τ*—shear stress (Pa), *K*—consistency coefficient (Pa·s^n^), $\dot{\gamma }$—shear rate (s^−1^), and *n*—flow behaviour index, *τ*_0_—yield stress (Pa).

*Oscillation stress sweep test:* the stress was increased from 0 to 300 Pa in 40 logarithmic steps at a constant frequency (1 Hz).

The frequency sweep test: frequency was increased from 0.01 to 30 Hz at 1Pa deformation fitting the range of the linear viscoelasticity.

### 4.9. Statistics

The statistical analysis involved a Statistica 12.5 (StatSoft, Tulsa, OK, USA) software employing the mono- and bifactorial analysis of variance and the Duncan’s test for checking the significance of the differences at *p* < 0.05.

For the microbiological tests, the statistical analysis was performed in Statistica v.13 (TIBCO Software, Palo Alto, Santa Clara, CA, USA). The descriptive statistics (mean, standard deviation, coefficient of variation) for the growth inhibition were calculated. A non-parametric Kruskal–Wallis test was applied to compare the effects of the Hyal/O_3_ and control foils, as well as the effect of Hyal/O_3_ against the different species of bacteria and Candida yeasts. The significance level for all tests was predetermined as *p* < 0.05.

### 4.10. Dynamic Light Scattering (DLS) and Zeta Potential—Analysis of the Hydrodynamic Diameter, Polydispersity and Particle Charge

For the research, we used the same original emulsions, which were used for the foil preparation. The emulsion was diluted 10 times in distilled water and left on a magnetic stirrer for 12 h until it was fully dispersed. The particle size dispersion and zeta potential were measured using a Zetasizer Nano Series ZS (Malvern, UK). In such a measurement, one experimental cycle equals at least 12 repetitions of the single analyses. For each sample, three full experimental cycles were performed and the average value from all experiments was analyzed.

### 4.11. Microbiological Analysis

#### 4.11.1. Determination of the Impact on the Microbial Growth

##### Isolation and Identification of the Tested Microorganisms

Swab samples were collected from various regions of the human skin and body, i.e., skin lesions of various types, the under eye region, cheeks, hands, back, eye, ear, mouth, throat, tonsils, vagina, anus. Sputum was also collected for the isolation of potentially pathogenic microorganisms. The samples were inoculated on general and selective media for the isolation of bacterial strains of the commensal skin microbiota, pathogens and potential pathogens as well as *Candida* species. Trypticase Soya agar (Biomaxima, Lublin, Poland) was used to isolate the bacterial members of the commensal skin microbiota (incubation for 24–48 h at 37 ± 1 °C), Mannitol Salt agar (Biomaxima, Lublin, Poland) was used for the isolation and preliminary identification of *Staphylococcus* spp. (yellow and pink colonies after incubation for 24–48 h at 37 ± 1 °C), Baird Parker agar was used for the isolation and identification of *Staphylococcus aureus* (grey to black colonies with clear halo after incubation for 24–48 h at 37 ± 1 °C), Columbia Agar with Sheep Blood Plus (Oxoid, Cheshire, UK) and UTI agar Plus (Oxoid, Cheshire, UK) were used for the isolation and identification of the type strains of *Escherichia coli* and *Staphylococcus aureus*, whereas Sabouraud Dextrose agar (Graso Biotech, Jabłowo, Poland), CandiSelect agar (Bio-Rad, Marnes-la-Coquette, France) were used for the isolation and preliminary identification of the *Candida*-type yeasts (incubation for 3–5 days at 35–37 ± 1 °C). Following the incubation and preliminary identification, the selected bacterial and yeast colonies were subcultured and subjected to microscopic observations of the Gram-stained preparations. The systematic position of the 53 bacterial strains was verified by MALDI-TOF (matrix-assisted laser desorption/ionization-time of flight) mass spectrometry and the systematic position of 30 yeast strains was determined using the CandiFast test kit (ELITechGroup, Puteaux, France).

### 4.12. Antimicrobial Activity of Hyaluronic Acid with Ozone

Antimicrobial activity of the examined preparations was assessed on a total of 83 microbial isolates. These included 53 bacterial isolates (four type strains and 49 isolates from the human skin) of 12 different species (Table 5) and 30 pathogenic *Candida* (two type strains and 28 isolates from various regions of the human body) of 12 different species (Table 6).

Microbial isolates were transferred to a sterile saline solution to obtain 0.5 MacFarland suspensions, then streaked onto Mueller–Hinton agar (Biomaxima, Lublin, Poland). Both sole Hyal and Hyal/O_3_ foils were sterilized under UV light for 30 min. Then, 10 × 10 mm squares were cut with a surface sterilized scalpel and applied onto the surface of the bacterial and yeast cultures. The cultures were incubated at 35 °C for 18–24 h (in case of bacteria) and for 3–5 days (in case of yeasts). Then, the results were read by observing whether the growth of the microorganisms was affected. In cases of growth inhibition, the diameters around the foil fragments were measured. Due to the fact that the applied foils were square, two diameters were read and the final result was expressed as a mean of the two reads (mm). All experiments were performed in three replications.

### 4.13. The Assessment of Cytotoxicity

The spontaneously immortalized human epidermal keratinocytes (HaCaT cell line) were seeded on a 96-well cell culture plate (1000 cells per well) in RPMI 1640 medium (Corning, USA), supplemented with fetal calf serum (EurX, Gdańsk, Poland) to a final concentration of 10%, 2 mM stable glutamine and antibiotic-antimycotic mixture: penicillin 50 I.U./mL, streptomycin 50 μg/mL and amphotericin B 250 ng/mL (from Biowest, Lo-Reninge, Belgium). Hyaluronic acid hydrogels (with or without ozonated olive oil) were diluted in the cell culture medium in 1:10, 1:25, 1:50 and 1:100 proportions, then 200 μL of these mixtures were added to each well with cells. The nontreated cells (NT control) received the standard cell culture medium. Following the 48 h incubation, the cell viability was determined, based on the ATP content of each well, using the luminometric Cell-Titer Glo test (Promega, Germany). The cell numbers were quantified, based on the calibration curve prepared with the known numbers of the HaCaT cells. Two independent experiments were performed, tetraplicates in each experimental group.

## 5. Conclusions

Nanocapsules of ozonated olive oil in hyaluronic acid, sized 50–100 nm, have been successfully obtained. The addition of ozonated olive oil nanocapsules did not significantly affect the structural changes of the polymer. The emulsions had a good rheological stability over time. The DLS study showed that hyaluronic acid in solutions without additives creates large flat structures, while when it interacts with the olive droplets, its structures are adapted to the shape and size of the olive droplet. The examined Hyal/O_3_ foils exhibited a very weak inhibitory effect against both bacterial commensal skin microbiota and pathogenic *Candida*-like yeasts. These results indicate that this formula can be treated as a safe ingredient of cosmetic preparations. A weak stimulatory effect of the Hyal+O_3_ foils on 17 out of the 53 bacterial strains (mainly *Micrococcus luteus*) is an umprecedented observation and requires further study. Both the Hyal and Hyal/O_3_ emulsions show a good biocompatibility and a lack of cytotoxicity in the HaCat keratinocyte model. The obtained results show that the Hyal/O_3_ composite is a promising material for use in medicine and cosmetology.

## Figures and Tables

**Table 1:** The parameters of Herschel–Bulkley rheological model.

Sample	Day of Measurement	Herschel–Bulkley Model				Area of Histeresis (Pa/s)
		K (Pa/s^n^)	n (−)	τ_0_ (Pa)	R^2^	
Hyal	1	6.88 ± 0.17 ^a^	0.42 ± 0.00 ^b^	3.21 ± 0.15 ^a^	0.9950	531.15 ± 52.68 ^a^
Hyal	15	5.59 ± 0.25 ^bc^	0.47 ± 0.04 ^ab^	2.13 ± 0.20 ^c^	0.9955	418.65 ± 0.21 ^b^
Hyal	30	4.85 ± 0.09 ^cd^	0.45 ± 0.0 ^ab^	2.32 ± 0.0 ^bc^	0.9955	385.95 ± 0.92 ^b^
Hyal/O_3_	1	6.35 ± 0.16 ^ab^	0.44 ± 0.00 ^ab^	2.57 ± 0.09 ^b^	0.9955	556.05 ± 21.28 ^a^
Hyal/O_3_	15	5.59 ± 0.87 ^bc^	0.44 ± 0.03 ^ab^	1.63 ± 0.1 ^d^	0.9953	582.32 ± 42.49 ^a^
Hyal/O_3_	30	4.11 ± 0.10 ^d^	0.48 ± 0.00 ^a^	1.33 ± 0.00 ^e^	0.9963	426.85 ± 8.56 ^b^

K—consistency coefficient, n—flow behaviour index, τ_0_—yield stress. Parameters in columns denoted with the same letters do not differ statistically at the level of confidence *p* < 0.05.

**Table 2:** Apparent viscosity of the gels at different shear rates.

Sample	Day of Measurement	Apparent Viscosity at Certain Shear Rates (Pa·s)			
		10 s^−1^	100 s^−1^	200 s^−1^	300 s^−1^
Hyal	1	1.64 ± 0.03 ^a^	0.53 ± 0.01 ^a^	0.34 ± 0.00 ^a^	0.26 ± 0.00 ^a^
Hyal	15	1.32 ± 0.06 ^c^	0.45 ± 0.01 ^b^	0.30 ± 0.01 ^b^	0.23 ± 0.00 ^b^
Hyal	30	1.19 ± 0.01 ^d^	0.42 ± 0.00 ^c^	0.28 ± 0.00 ^c^	0.21 ± 0.00 ^c^
Hyal/O_3_	1	1.51 ± 0.03 ^cd^	0.52 ± 0.01 ^a^	0.34 ± 0.01 ^a^	0.26 ± 0.00 ^a^
Hyal/O_3_	15	1.24 ± 0.07 ^b^	0.46 ± 0.01 ^b^	0.30 ± 0.01 ^b^	0.23 ± 0.00 ^b^
Hyal/O_3_	30	1.00 ± 0.03 ^e^	0.41 ± 0.01 ^c^	0.28 ± 0.00 ^c^	0.22 ± 0.00 ^c^

Parameters in the columns denoted with the same letters do not differ statistically at the level of confidence *p* < 0.05.

**Table 3:** Number of commensal bacterial strains, the growth of which was increased, as a result of the Hyal/O_3_ foil application.

	Origin			
Species	Under Eyes	Cheeks	Hands	Total
*S. aureus*	1	0	0	1
*S. haemolyticus*	1	1	2	4
*S. epidermidis*	1	0	1	2
*M. luteus*	2	6	2	10
Total	5	7	5	17

**Table 4:** Growth inhibition zones for the bacterial and Candida strains, as a result of the Hyal/O_3_ foil application (mm).

Species	Origin	Hyal+O_3_	C (Hyal)
*S. aureus* MSSA ATCC 29213	type strain	17	0
*S. aureus* MRSA NCTC 12492	type strain	19	0
*M. luteus* 1	hands	13	0
*M. luteus* 2	hands	12	0
*A. viridans*	hands	14	0
mean		15.07	
Standard deviation		3.01	
Coefficient of variation (%)		19.99	
*C. parapsilosis* 1	skin lesions	8	0
*C. parapsilosis* 2	throat	17	0
*C. albicans*	mouth	10	0
*C. guillermondii*	eye	9	0
*C. glabrata*	vagina	10	0
mean		10.93	
Standard deviation		5.49	
Coefficient of variation (%)		50.26	

**Table 5:** Characteristics of the bacterial—commensal strains used in the analysis.

	Origin			
Species	Under Eyes	Cheeks	Hands	Type Species
*Staphylococcus aureus*	2	2	2	*S. aureus* MSSA ATCC 29213 *S. aureus* MRSA NCTC 12492
*S. haemolyticus*	2	5	3	
*S. epidermidis*	1	1	3	
*S. saprophyticus*	0	1	1	
*S. warneri*	0	1	0	
*S. cohnii*	0	0	1	
*S. pasteuri*	0	0	2	
*Micrococcus luteus*	5	7	5	
*Kocuria palustris*	0	1	1	
*Bacillus megaterium*	0	0	1	
*Aerococcus viridans*	0	0	1	
*Pseudomonas fulva*	0	0	1	
*Escherichia coli*	0	0	0	*E. coli* ESBL+
Total	10	18	21	4

**Table 6:** Characteristics of the pathogenic Candida yeast strains used in the analysis.

	Skin Lesions	Eye	Mouth/Throat/Tonsils	Vagina/Anus	Sputum	Type Species
*C. parapsilosis*	3	0	1	0	0	*Candida parapsilosis* ATCC 22019
*C. albicans*	1	0	2	1	0	*Candida albicans* ATCC 90028
*C. inconspicua*	1	0	2	1	0	
*C. guillermondii*	1	2	0	0	0	
*C. famata*	0	0	1	0	0	
*C. glabrata*	0	0	0	2	1	
*C. lusitaniae*	0	0	0	3	0	
*C. krusei*	0	0	0	2	1	
*C. lambica*	0	0	0	1	0	
*C. tropicalis*	0	0	1	0	0	
*C. kefir*	0	0	1	0	0	
Total	6	2	8	10	2	2

## Data Availability

The data presented in this study are available upon request from the corresponding author.
